# Breastfeeding and biomarkers of folate and cobalamin status in Norwegian infants: a cross-sectional study

**DOI:** 10.1017/jns.2024.54

**Published:** 2024-09-19

**Authors:** Beate S. Solvik, Kjersti S. Bakken, Adrian McCann, Per M. Ueland, Sigrun Henjum, Tor A. Strand

**Affiliations:** 1 Innlandet Hospital Trust, P.O. Box 990, Lillehammer 2629, Norway; 2 Centre for International Health, University of Bergen, P.O. Box 7800, Bergen 5020, Norway; 3 Bevital AS, Bergen, Norway; 4 Department of Nursing and Health Promotion, Faculty of Health Sciences, OsloMet – Oslo Metropolitan University, Norway

**Keywords:** Breastfeeding, Folate, Homocysteine, Methylmalonic acid, Vitamin B_12_

## Abstract

Folate and vitamin B_12_ (cobalamin) are essential for growth and development. This cross-sectional study aims to describe folate and vitamin B_12_ status according to infant age and breastfeeding practices in Norwegian infants. Infants aged 0–12 months (*n* = 125) were recruited through public health clinics. We registered breastfeeding status and measured serum concentrations of folate, cobalamin, total homocysteine (tHcy), and methylmalonic acid (MMA). The associations between infant age, breastfeeding, and biomarker concentrations were estimated in regression models. The mean (SD) age was 24 (16) weeks, and 42% were exclusively breastfed, 38% were partially breastfed, and 21% were weaned. Overall, median (IQR) folate, cobalamin, tHcy, and MMA concentrations were 47 (35–66) nmol/L, 250 (178–368) pmol/L, 6.99 (5.69–9.27) µmol/L, and 0.35 (0.24–0.83) µmol/L, respectively. None of the infants were folate deficient, 15% were vitamin B_12_ deficient (< 148 pmol/L), and 23% had low vitamin B_12_ status (148–221 pmol/L). Elevated tHcy (> 6.5 μmol/L) and MMA (> 0.26 μmol/L) were found in 62% and 69% of the infants, respectively. Compared to weaned, exclusively or partially breastfed infants were younger and had 46% higher tHcy concentrations (*P* < 0.001), in addition to 47% and 39% lower cobalamin concentrations (*P* < 0.001), respectively. However, the observed biomarker concentrations appeared to be independent of infant age. In conclusion, low vitamin B_12_ status was prevalent and appeared to be more common in the younger exclusively breastfed compared to older weaned infants. The implications of low vitamin B_12_ status in infancy are unknown and require further investigation.

## Introduction

Folate and vitamin B_12_ (cobalamin) play pivotal roles in DNA synthesis, cell division, formation of red blood cells, and myelination of the nervous system.^(1)^ Due to rapid growth and development, these vitamins are especially important during infancy.^(2)^ Infant dietary sources of folate and vitamin B_12_ include breast milk, formula milk, and complementary foods. Even though exclusive breastfeeding is important and recommended during the first 4–6 months of life,^(3)^ breast milk is not a nutritionally complete food due to low concentrations of vitamin D and K.^(4)^ Breast milk is considered a good source of folate;^(4)^ however, concerns have been raised about low vitamin B_12_ concentrations in breast milk, especially in the context of exclusively and prolonged breastfeeding.^(5,6)^

The vitamin B_12_ concentration of breast milk depends on maternal status and potentially maternal vitamin B_12_ intake during pregnancy and lactation.^(7)^ The vitamin B_12_ content in breast milk declines during the first 4–6 months of lactation.^(5,8,9)^ A declining infant vitamin B_12_ status from birth to 4–6 months of age has been observed.^(5,10)^ In these studies, formula-fed or partially breastfed infants had a biomarker profile indicative of a more favorable vitamin B_12_ status, compared to exclusively breastfed.^(5,10)^ Further, studies in both high- and low-income settings suggest that breastfed infants across different age groups appear to have lower cobalamin concentrations, accompanied by higher circulating concentrations of the functional biomarkers of vitamin B_12_ status, total homocysteine (tHcy), and methylmalonic acid (MMA), compared to non-breastfed infants.^(11–14)^

Typical symptoms of infant vitamin B_12_ deficiency are failure to thrive, lethargy, hypotonia, food refusal, and cognitive and motor developmental delays.^(15)^ Low vitamin B_12_ status (often defined as serum cobalamin 148–221 pmol/L) is typically asymptomatic but may adversely affect long-term growth and development.^(1)^ For instance, vitamin B_12_ status during infancy was associated with impaired growth and performance on neuropsychological tests in children at 5 years of age, even though only a small percentage of these children were classified as having vitamin B_12_ deficiency (< 148 pmol/L) during infancy.^(16,17)^

Various biomarkers, cut-offs, and algorithms have been used to define deficiency and low vitamin B_12_ status; however, limited age-specific reference intervals and cut-off values exist.^(18)^ In this study, we describe maternal dietary intake of vitamin B12 and investigate the direct biomarkers serum folate and cobalamin in conjunction with the functional biomarkers, MMA and tHcy, in Norwegian infants. Interpretation of these markers remains challenging because of the relation between folate and tHcy, and other factors impacting the utility of MMA. For these reasons and in an effort to improve the biochemical assessment of vitamin B_12_ status, we also included the combined indicator of vitamin B_12_ status, 3cB_12_.^(19)^ Furthermore, we aimed to evaluate the correlation between these biomarkers and explore their association with infant age and breastfeeding status.

## Methods

### Study procedure and participants

We examined data from a cross-sectional study investigating iodine status conducted at two public health clinics in Norway. Mothers, who had a scheduled visit to the clinics and whose infants were aged 0–12 months, were asked to participate. Mother–infant pairs were recruited from October to December 2018. The study design, including dietary assessment and eligibility criteria, have previously been described in detail.^(20,21)^ In brief, information regarding the participants’ background, breastfeeding status, and diet was collected during structured iodine-based 24 h dietary recalls and via online questionnaires that contained a food frequency questionnaire (FFQ). The FFQ was not validated. Prior to the start of the study, the mothers provided written informed consent. The trial was approved by the Regional Committees for Medical and Health Research Ethics South-East Norway (2018/1230). All study procedures were performed in accordance with the Declaration of Helsinki.

### Dietary intake

Maternal habitual vitamin B_12_ was estimated from an FFQ containing 31 food items. The FFQ contained questions regarding the consumption of vitamin B_12_-containing food groups such as meat (red, white, and game), fish (fat and lean), dairy products (milk, yoghurt, and cheese), and egg during the preceding 4 weeks. Each food group had seven possible responses: rarely/never, less than weekly, 1–3 times weekly, 4–6 times weekly, 1–2 times daily, 3–4 times daily, and 5 times daily or more. The frequencies were converted into daily amounts. The dietary intake of vitamin B_12_ was calculated using the Norwegian Food Composite Table^(22)^ and standard portion sizes^(23)^ or portion sizes as defined in the FFQ. We also calculated the intake of vitamin B_12_ from supplements using data from the structured iodine-based 24 h dietary recall. In this question, we captured the name and dosage of the supplements, in addition to how often the supplements were consumed per week. The same questions were used to capture infant’s consumption of supplements. Information regarding the infant’s consumption of formulas and cereals was collected from FFQs and 24 h dietary recalls. The nutrient content of reported supplements, formulas and cereals was based on information from the producers.

### Biochemical analyses

Heel capillary blood samples were collected by a trained and dedicated staff in 1.5 mL micro tubes (Sarstedt AG & Co.) and left at room temperature for approximately 15 min before centrifuged (2000 G, 20°C, 10 min). The serum samples were stored at −80°C and transported on dry ice for analyses. We used microbiological assays based on a chloramphenicol-resistant strain of *Lactobacillus casei* ^(24)^ and colistin sulphate-resistant strain of *Lactobacillus leichmannii*,^(25)^ to determine serum folate and cobalamin concentrations. Serum concentrations of MMA and tHcy were analysed by gas chromatography-tandem mass spectrometry (GC-MS/MS) based on methylchloroformate derivatisation.^(26)^ The within- and between-day coefficient of variation was 4% and 5%, respectively, for both folate and cobalamin. For MMA and tHcy, within- and between-day coefficient of variation ranged from 1 to 5% and 1 to 3%, respectively.^(26)^ All blood samples were analysed at Bevital Laboratory, Bergen, Norway (www.bevital.no).

### Definitions and cut-off values

There is a lack of consensus on age-specific reference values for defining folate and cobalamin deficiency. Therefore, we used the World Health Organization (WHO) following cut-offs, previously defined for adults: serum folate concentrations of > 45.3 nmol/L, < 13.4 nmol/L, and < 6.8 nmol/L were considered as ‘elevated’, ‘possible deficiency’, and ‘deficiency’, respectively.^(27)^ We also used the WHO-suggested cut-off of < 10 nmol/L to define folate deficiency.^(27)^ Cobalamin concentrations of < 148 pmol/L, 148–221 pmol/L, and < 250 pmol/L were defined as ‘deficiency’, ‘low vitamin B_12_’, and ‘subclinical deficiency’, respectively.^(18)^ In addition to the direct measures of folate and cobalamin status, we used the functional biomarkers MMA and tHcy. For adults, MMA concentrations above 0.26 μmol/L and tHcy concentrations above 13 μmol/L have been considered elevated.^(18)^ In addition, we used a tHcy cut-off of > 6.5 μmol/L as a metabolic indicator of low vitamin B_12_ status in infants, as previously proposed.^(28)^ This cut-off denoted the 97.5th percentile of infants following an intramuscular cobalamin injection^(28)^ and has also been used in two RCTs evaluating the effect of intramuscular cobalamin injection on cognitive and motor function.^(11,29)^ While vitamin B_12_ status is assessed predominantly by total serum cobalamin concentrations, both the sensitivity and specificity of the marker are questionable which is why the functional markers MMA and tHcy also tend to be measured. We also included the combined indicator of vitamin B_12_ status, 3cB_12_. In brief, the 3cB_12_ is calculated by the log-transformed cobalamin over the product of tHcy and MMA concentrations. In adults, a score of < −0.5 is regarded as low vitamin B_12_ status.^(19)^ Self-reported maternal height and weight (at inclusion) were used to calculate the body mass index (BMI) in kg/m^2^. We stratified maternal BMI into four categories: underweight (< 18.5 kg/m^2^), normal weight (18.5–24.9 kg/m^2^), overweight (25–29.9 kg/m^2^), and obese (> 30 kg/m^2^). The birth weight was stratified into three categories: low birth weight (< 2500 g), normal birth weight (2500–4500 g), and high birth weight (> 4500 g). Breastfeeding status was categorised as exclusively breastfed (only breast milk and vitamins/minerals), partially breastfed (defined as breast milk and other liquids, or breast milk and formulas, or breast milk and complementary food, or a combination of breast milk and liquids and complementary foods) and weaned infants (including 2 infants that were never breastfed). The infants were categorised based on current breastfeeding status as reported by the mothers in the 24 h dietary recalls and FFQs.

### Statistical methods

Binary and categorical variables were reported as frequencies and percentages (infant gender, preterm delivery, birth weight, marital status, educational attainment, maternal BMI, maternal dietary preferences, breastfeeding status, and supplement use). Maternal and infant’s age were reported as mean (SD). Biomarker concentrations were reported as median and interquartile range (IQR) due to the skewed distribution of the data. The biomarkers were also enumerated (frequency and percentages) according to suggested cut-off values. Multiple linear regression models were used to explore the possible associations between biomarker concentrations (log-transformed concentrations of folate, cobalamin, MMA, and tHcy and 3cB_12_), the infant’s age, and breastfeeding status. Spearman’s rank-order correlation coefficient was used to describe the monotonic correlations between the different biomarkers. We used fractional-polynomial prediction plots to explore non-linear relationships between the biomarkers. In these plots, we removed the lowest observed cobalamin concentrations. We also made a prediction plot of biomarker concentration as a function of age, according to breastfeeding status (exclusively or partially breastfed versus weaned/never breastfed). For interpretation of this plot, we included only infants above 6 weeks of age, so the curves (breastfed versus weaned) had the same starting point. Statistical analyses were performed using the statistical software Stata, version 16 (STATA Corp, Houston, TX, USA). *P* values < 0.05 were considered statistically significant.

## Results

### General characteristics

**Fig. 1.**
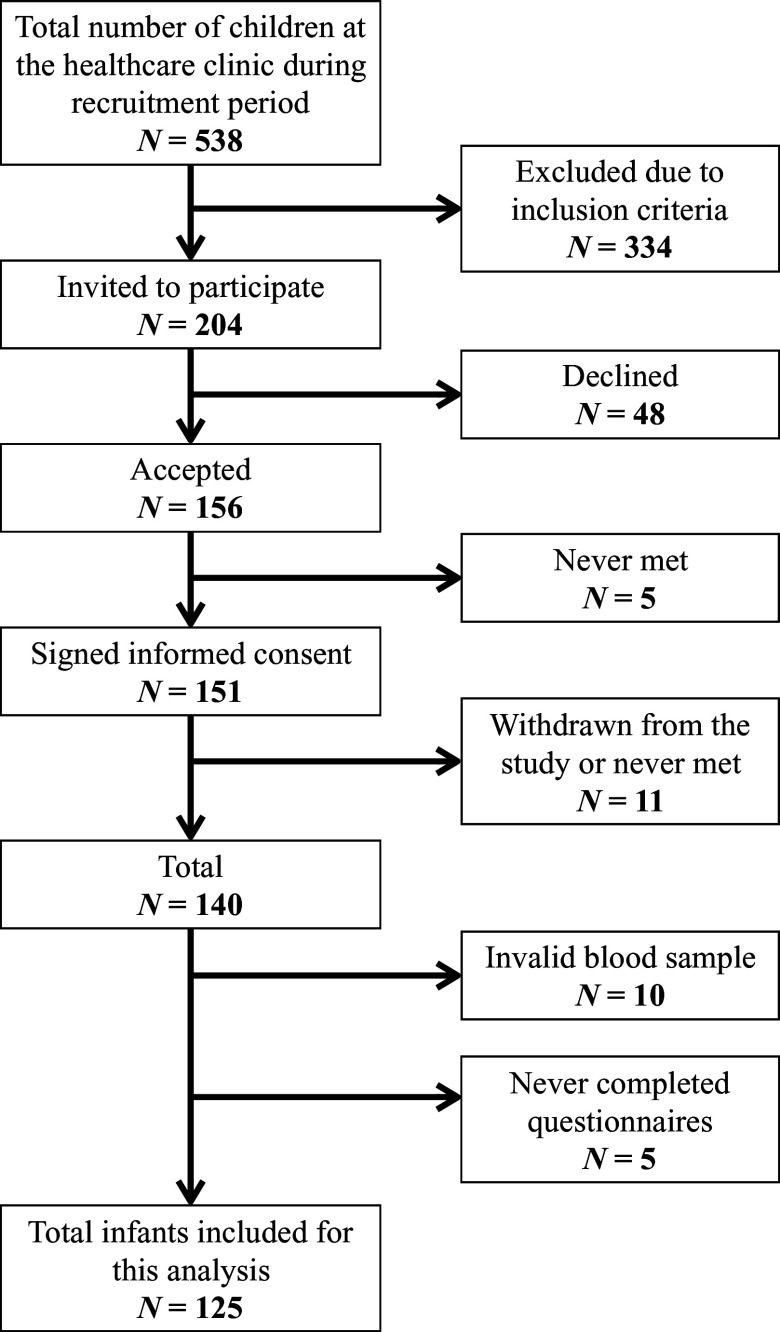
Flow chart of participants.

A total of 151 mother–infant pairs were recruited from the public health clinics. Eleven mother–infant pairs withdrew from the study or never met for sampling. Blood samples were available from 125 remaining infants (Fig. 1). The mother–infant pairs’ background information is summarised in Table 1. Five infants had low birth weight (< 2500 g) and no infant had a birth weight under 2000 g.

**Table 1.** Baseline characteristics of 125 Norwegian mother–infant pair^a^

Characteristic	Total (*n* = 125)	Exclusive (*n* = 52)	Partial (*n* = 47)	Weaned (*n* = 26)
Age child, week	24 ± 16	11 ± 6	31 ± 12	37 ± 15
Age mother, years	31 ± 5	31 ± 4	31 ± 5	31 ± 4
Girls	61 (49)	23 (44)	26 (55)	12 (46)
Preterm delivery^b^	17 (14)	5 (10)	8 (17)	4 (15)
Birth weight				
< 2500 g	5 (4.0)	1 (1.9)	2 (4.5)	2 (7.7)
2500–4500 g	116 (93)	49 (94)	44 (94)	23 (88)
> 4500 g	4 (3.2)	2 (3.8)	1 (2.1)	1 (3.8)
Marital status				
Single	1 (0.8)	0 (0)	1 (2.2)	0 (0)
Cohabitation	75 (61)	33 (65)	31 (69)	11 (42)
Married	46 (38)	18 (35)	13 (29)	15 (58)
Education, mother				
< 12 years	4 (3.2)	1 (1.9)	1 (2.2)	2 (7.7)
12 year	19 (15)	7 (13)	9 (20)	3 (12)
1–4 years college/university	47 (38)	20 (38)	14 (30)	13 (50)
> 4 years college/university	54 (43)	24 (46)	22 (48)	8 (31)
Maternal BMI at inclusion				
< 18.5 (underweight)	2 (1.7)	0 (0)	1 (2.3)	1 (4.3)
18.5–24.9 (normal weight)	77 (66)	34 (67)	30 (70)	13 (57)
25–29.9 (overweight)	25 (21)	13 (25)	7 (16)	5 (22)
> 30 (obese)	13 (11)	4 (7.8)	5 (12)	4 (17)
Maternal dietary variables				
Vegetarian/vegan	3 (2.4)	0 (0)	2 (4.4)	1 (3.8)
Vitamin B supplements^c^	38 (32)	17 (32)	12 (26)	9 (35)
Infant dietary intake^d^				
Formula	28 (22)	0 (0)	10 (21)	18 (69)
Fortified cereals	47 (38)	0 (0)	28 (60)	19 (73)
Vitamin B_12_ fortified cereals	32 (26)	0 (0)	18 (38)	14 (54)
Non-fortified cereals	8 (6)	0 (0)	5 (11)	3 (12)
Serum biomarkers				
Folate, nmol/L	47.2 (34.5, 65.8)	39.3 (30.0, 62.6)	48.9 (38.8, 65.6)	61.7 (46.4, 85.8)
Cobalamin, pmol/L	250 (178, 368)	223 (167, 277)	239 (190, 315)	391 (348, 461)
tHcy, µmol/L	6.99 (5.69, 9.27)	7.43 (6.19, 9.59)	7.22 (6.29, 9.34)	4.94 (4.52, 6.23)
MMA, µmol/L	0.35 (0.24, 0.83)	0.33 (0.26, 1.01)	0.67 (0.35, 1.20)	0.21 (0.17, 0.25)
3cB12	−0.32 (−1.05, 0.23)	−0.38 (−1.38, − 0.10)	−0.74 (−1.35, −0.11)	0.47 (0.23, 0.77)

tHcy, total homocysteine; MMA, methylmalonic acid; 3cB12, combined indicator of vitamin B_12_ status including 3 biomarkers (cobalamin, MMA, and tHcy). ^a^Values are mean ± SD, median (IQR), or *n* (%). ^b^Defined as birth < 37 weeks of gestation. ^c^Reported as weekly intake from 24 h dietary recall. ^d^Reported as daily intake from food frequency questionnaires.

### Dietary intake

Fortified cereals were consumed by 60% of the partially breastfed infants and 73% of the weaned infants. All fortified cereals were enriched with folic acid, and 38% of partially breastfed and 54% of weaned infants consumed cereals additionally enriched with vitamin B_12_. Among the partially breastfed infants, 17% of the infants had not been introduced to complementary foods and were only consuming formulas in addition to breast milk. For the weaned and partially breastfed infants, the mean age (SD) for introduction to complementary foods was 4.5 (0.74) months. Only one child consumed a multimicronutrient supplement and another child consumed a folic acid supplement. Among the mothers, 38 (32%) reported consuming supplements of folate, vitamin B_12_, or a combination of these vitamins. Most of the reported supplements contained multiple micronutrients. The daily content of folic acid and vitamin B_12_ in these supplements ranged from 75 to 400 µg and 1 to 10 µg, respectively. Two mothers were consuming high-dose vitamin B_12_ supplements that contained 500 µg. One mother was vegetarian, and two mothers were vegans. Median (IQR) dietary intake of vitamin B_12_ was 4.2 (3.0–5.5) µg from food and 4.6 (3.5–6.3) µg from foods and supplements combined. Among the lactating women, 91% met the recommended intake (RI) from 2012 of 2.6 µg/d,^(30)^ while 63% met the newly suggested adequate intake (AI) of 4.2 µg/d.^(31)^ For the non-lactating women, 81% met the RI from 2012 of 2.0 µg/d,^(30)^ while 58% met the newly suggested AI of 4.0 µg/d.^(31)^

### Infant folate and vitamin B_12_ status

**Fig. 2.**
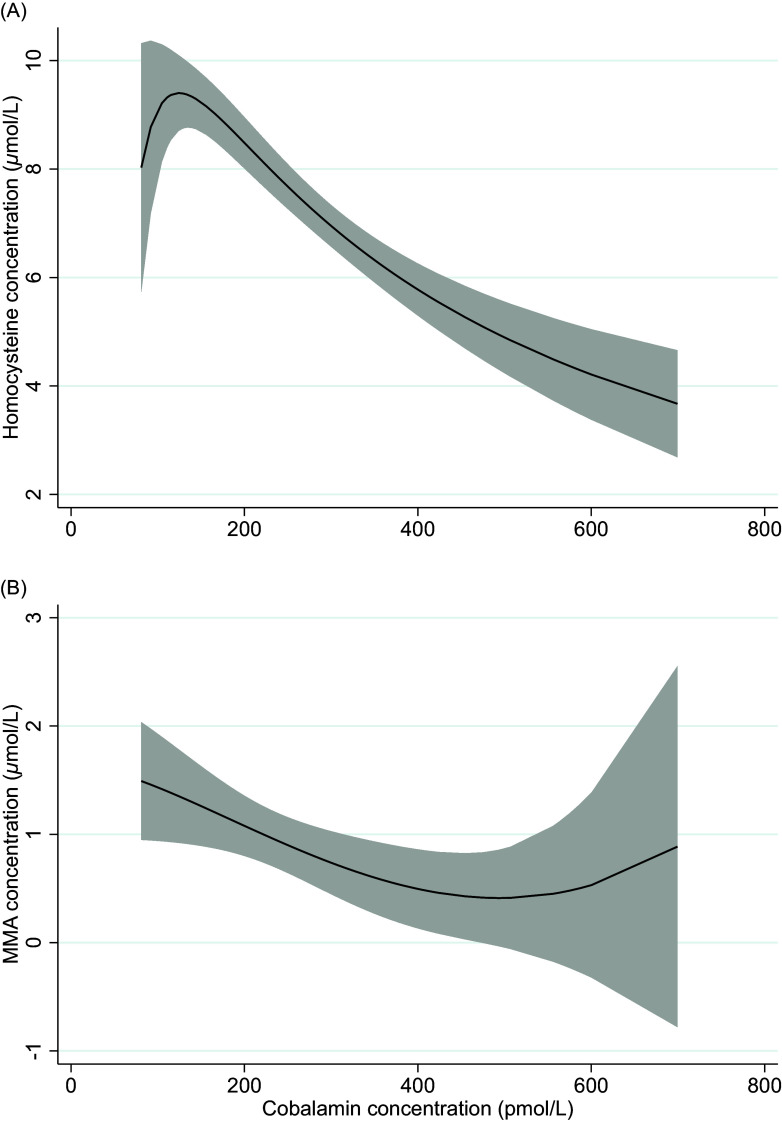
Two-way fractional-polynomial prediction plot describing the correlation between serum concentrations of cobalamin and homocysteine (panel A) and cobalamin and methylmalonic acid (panel B).

The median (IQR) concentrations of direct and functional biomarkers of folate and cobalamin status are reported in Table 1. Suggested cut-off values and the respective prevalence are shown in Table 2. Folate deficiency was not present among any of the infants (regardless of the cut-off values used). Vitamin B_12_ deficiency (cobalamin < 148 pmol/L) was identified in 10 exclusively breastfed, eight partially breastfed, and one weaned infant. None of the weaned infants had low vitamin B_12_ status (cobalamin 148–221 pmol/L). According to the 3cB_12_ cutoff used in adults (3cB_12_ < −0.5), 42.2% infants had low vitamin B_12_ status. Serum cobalamin was inversely correlated with serum concentrations of tHcy (rho = −0.67, *P* < 0.001) and MMA (rho = −0.51, *P* < 0.001). These correlations are depicted in Fig. 2.

**Table 2.** Suggested cut-off values and corresponding prevalence of vitamin B_12_ and folate deficiency among 125 Norwegian infants^a^

Cut-off	Definition ^(ref)^	*n* (%)
Folate < 13.4 nmol/L	Possible deficiency^(27)^	0 (0.0)
Folate < 10 nmol/L	Deficiency^(27)^	0 (0.0)
Folate < 6.8 nmol/L	Deficiency^(27)^	0 (0.0)
Folate > 45.7 nmol/L	Elevated Folate^(27)^	65 (52.0)
Cobalamin < 148 pmol/L	Deficiency^(18)^	19 (15.2)
Cobalamin 148–221 pmol/L	Low vitamin B_12_^(18)^	29 (23.2)
Cobalamin < 250 pmol/L	Subclinical deficiency^(18)^	63 (50.4)
tHcy > 13 µmol/L	Elevated^(18)^	5 (4.0)
tHcy > 6.5 µmol/L	Elevated^(28)^	78 (62.4)
MMA > 0.26 µmol/L	Elevated^(18)^	87 (68.8)
3cB12 < −0.5	Low status^(19)^	53 (42.4)

tHcy, total homocysteine; MMA, methylmalonic acid; 3cB_12_, combined indicator of vitamin B_12_ status including 3 biomarkers (cobalamin, MMA, and tHcy). ^a^Values are *n* (%). References for the cut-offs values and definitions included in parentheses.

### Infant age and breastfeeding status and their association with folate and cobalamin status

**Fig. 3.**
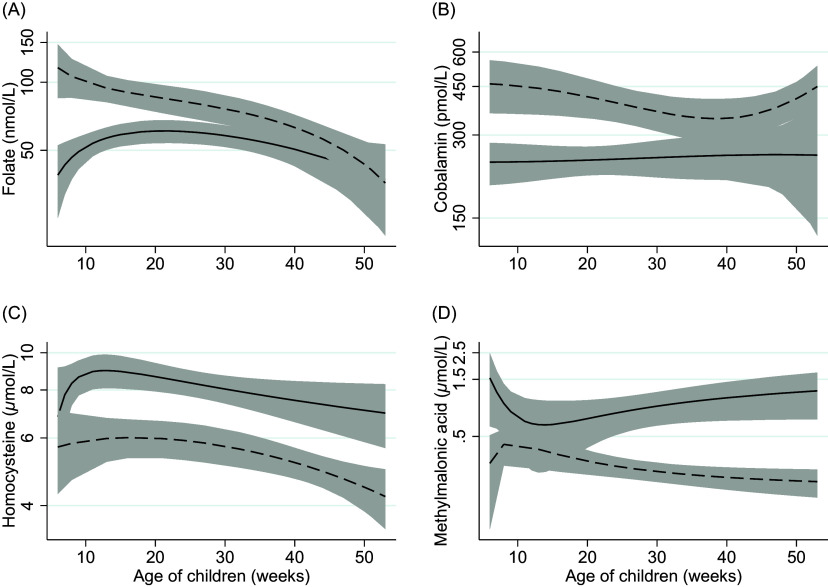
Two-way fractional-polynomial prediction plot describing the correlation between serum concentrations of folate (panel A), cobalamin (panel B), homocysteine (panel C), methylmalonic acid (panel D), and age of infants (weeks), according to breastfeeding status. *Footnotes*: The solid line represents breastfed (exclusive and partially) infants (*n* = 84). The dashed line represents weaned and never breastfed infants (*n* = 26). The grey area shows the 95% CI.

Results from the regression analyses are reported in Table 3. Although none of the included infants were folate deficient, infants that were exclusively breastfed had 35% (*P* = 0.004) lower serum folate concentrations compared to weaned infants. The difference in folate concentration between partially breastfed and weaned infants did not reach statistical significance (*P* = 0.08). Compared to weaned infants, exclusively or partially breastfed infants had a 47% and 39% lower cobalamin concentration (*P* < 0.001), respectively. Both exclusively and partially breastfed infants also had a 46% higher serum tHcy concentration (*P* < 0.001). Serum MMA concentrations were more than two times higher in partially breastfed compared to weaned infants (*P* < 0.001). The mean difference in MMA concentration between exclusively breastfed and weaned infants did not reach statistical significance (*P* = 0.186). Compared to weaned infants, the 3cB12 scores were 0.84 and 1.17 units lower in exclusively and partially breastfed infants, respectively. The infant’s age was not associated with biomarker concentrations. Figure 3 depicts biomarker concentrations in relation to infant age according to breastfeeding status.

**Table 3.** Generalised linear models for the associations between breastfeeding status and infants’ age in relation to biomarkers of vitamin B_12_ status^a^

	Folate^b^	Cobalamin^b^	tHcy^b^	MMA^b^	3cB_12_^c^
Breastfeeding status					
Never/Weaned	Ref	Ref	Ref	Ref	Ref
Partially	0.82 (0.65, 1.02)	0.61 (0.50, 0.75)	1.46 (1.27, 1.69)	2.84 (1.88, 4.29)	−0.84 (−1.31, −0.37)
Exclusively	0.65 (0.49, 0.87)	0.53 (0.41, 0.69)	1.46 (1.21, 1.76)	1.43 (0.84, 2.44)	−1.17 (−1.53, −0.80)
Age of infant, week	1.00 (0.99, 1.01)	1.00 (0.99, 1.00)	1.00 (0.99, 1.00)	0.99 (0.97, 1.00)	0.008 (−0.005 to 0.02)

tHcy, total homocysteine; MMA, methylmalonic acid. 3cB_12_, combined indicator of vitamin B_12_ status including 3 biomarkers (cobalamin, MMA, and tHcy). ^a^ *n* = 125. ^b^The values of the single biomarkers were log-transformed and the regression coefficients exponentiated (95% CIs). Thus, the effect measures represent the relative change from the reference group, i.e. the concentration of folate is 18% lower among the partially breastfed infants and 35% lower among the exclusively breastfed infants compared to the reference (never/weaned). ^c^Regression coefficients (95% CIs) using untransformed 3cB_12_.

## Discussion

A key finding of the current study was the low vitamin B_12_ status in breastfed infants compared to weaned infants, independent of infant age (Fig. 3). Differences in vitamin B_12_ status according to infant’s feeding practice have previously been reported worldwide^(12–14,32)^ and among Norwegian infants^(10,11)^ and are now replicated in the present study. Notably, the Norwegian studies have been conducted over the previous 10–25 years, and given the recent focus on and recommendation of more plant-based diets (typically with reduced vitamin B_12_ availability), our findings underscore the importance of evaluating vitamin B_12_ status in certain groups, such as infants.

### Infant’s feeding practice

The vitamin B_12_ concentration in breast milk depends on maternal biochemical status, both during pregnancy and after,^(7)^ which unfortunately was not assessed in the present study. The partially breastfed infants also had lower serum cobalamin concentrations alongside higher serum tHcy compared to weaned infants. Similar findings have previously been reported among 6 and 12 months old Norwegian infants.^(10)^ This may be related to low dietary cobalamin intake despite the introduction of complementary foods. Fruits and vegetables, which do not contain vitamin B_12_, are common weaning foods,^(33)^ while dairy, the food group that typically contributed the most to vitamin B_12_ intake among Norwegian toddlers,^(34)^ is not recommended before 10–12 months of age.^(35)^ Industrialised porridge is another very common complementary food.^(33)^ Some, but not all, industrialised porridge on the Norwegian market is fortified with vitamin B_12_. Other studies have found associations between infant age and vitamin B_12_ status;^(36,37)^ however, we were not able to find such associations in the analyses taking feeding into account.

### Maternal dietary vitamin B_12_ intake

Our estimation of maternal dietary vitamin B_12_ intake was lower than that reported in the Norwegian Mother and Child Cohort (Moba) where the mean (SD) dietary intake (including supplements) of vitamin B_12_ was 8.8 (23.2) µg/d.^(38)^ A plausible explanation for the higher vitamin B_12_ estimations in Moba is the use of a more comprehensive FFQ containing a total of 250 foods, compared to our 31 foods. Thus, the estimated dietary intake of vitamin B_12_ in the present study might be too low, and the percentage of lactating women meeting the adequate intake of vitamin B_12_ is likely underestimated. The vitamin B_12_ concentration in breast milk is considered to depend on maternal status,^(4)^ and in two randomised controlled trials (RCT), maternal vitamin B_12_ supplementation intake improved breast milk concentration and infant vitamin B_12_ status.^(39,40)^ However, in these RCTs, the supplementation started in early pregnancy and continued until 6–12 weeks postpartum. In addition, the supplement contained high doses of vitamin B_12_ (50–250 µg/d). Unfortunately, we do not have information regarding maternal dietary intake and supplement use during pregnancy, which potentially could have an impact on the infants’ vitamin B_12_ status, particularly in early infancy.

### Cobalamin and tHcy concentration

In general, the infants’ serum cobalamin concentrations in our study are reasonably comparable to other studies among newborns and infants in high-income settings,^(5,9,37,41–43)^ as well as in some middle- and low-income settings.^(36,44)^ Likewise, the tHcy concentrations observed in our study were comparable to other Scandinavian studies,^(9,37,41,42,45)^ but not with studies from low-income settings. For instance, in Nepal, the infants’ plasma tHcy concentrations were almost twice that of tHcy observed in our study, even though infants from these populations had comparable circulating cobalamin and folate measurements.^(36,44)^ Other studies have shown that genetic variations, BMI, and exposure to air pollution may influence the tHcy concentrations.^(46,47)^ Despite these shortcomings, we and others have found that tHcy is a good marker of infant vitamin B_12_ status.^(42,44,45)^

### MMA concentration and 3cB_12_

The infants’ cobalamin concentrations were also correlated to MMA, but less strongly. This finding is consistent with other studies among infants.^(42,45)^ Even though serum cobalamin concentrations in exclusively and partially breastfed infants were similar, serum MMA concentrations were higher in partially breastfed infants. The higher MMA concentrations in partially breastfed infants may be related to factors other than cobalamin status, such as increased synthesis or dietary intake of MMA precursors. A number of metabolic pathways, including pyrimidine and propionic acid metabolism as well as branched amino acid (BCAA) degradation, converge upstream of MMA. It is therefore feasible that increased intake of BCAAs or synthesis of propionic acid (produced by the microbiome) stemming from ingestion of formula milk and/or introduction of complementary foods may affect the serum MMA concentrations observed among the partially breastfed infants in the current study. Compared to formula milk, breast milk has lower BCAA content and breastfed infants have lower plasma concentrations of BCAA compared to formula-fed infants.^(48)^ Evolving microbiota represents another explanation and is likely modified by the introduction of complementary foods.^(49)^ Colonisation of certain bacteria may increase the production of MMA precursors such as propionate or odd-chain fatty acids.^(50)^ Such mechanisms potentially undermine the specificity and utility of MMA as a biomarker of vitamin B_12_ status, both alone and as a component of a combined indicator, especially for infants. For instance, the higher MMA concentrations in partially breastfed infants could also explain the lower 3cB_12_ observed in this group. Few, but some other studies have used 3cB_12_ in infants.^(17,51)^ However, caution should be urged when evaluating 3cB_12_ in infants given that the algorithm was developed in adults over 18 years of age.^(19)^ Moreover, we did not measure holotranscobalamin (holo-TC), which was originally included in the complete combined indicator of vitamin B_12_ status (4cB_12_).^(19)^ Our observations suggest the validity of the combined indicator may be limited by the number of, and which biomarkers are used when evaluating vitamin B_12_ status among infants. It is also worth noting that the use of a four-marker combined indicator tends to be precluded in both research and clinical settings which routinely assess only 2–3 vitamin B_12_-related markers due to costs and other logistical considerations.

### Folate concentration

None of the infants were observed to have deficient or low folate status, although exclusively breastfed infants had lower serum folate concentrations compared to weaned infants. This is surprising given that breast milk is considered a good source of folate.^(4)^ One possible explanation could be a high dietary intake of folate (fruits, vegetables, and liver) or folic acid (formulas and industrialised cereals). Another explanation is that folic acid from formulas and enriched cereals has a higher bioavailability than folate.^(52)^ The high serum folate concentration could also be due to low serum cobalamin concentrations. This could be explained by a phenomenon commonly known as the ‘methylfolate trap’.^(53)^ This phenomenon is explained by poor function of the vitamin B_12_-dependent enzyme methionine synthase, resulting in an artificially elevated 5-methyltetrahydrofolate concentration, masking true folate status. Moreover, we did not find any correlation between circulating folate and tHcy concentrations, which contrasts with findings among older Norwegian children.^(54,55)^ Besides limited statistical power, another likely explanation for the absence of correlation between folate and tHcy is the adequate folate status among infants in the current study.

### Cut-off values and consequences of low vitamin B_12_ status

As previously stated, there are no age-specific cobalamin cut-off values for assessing infant vitamin B_12_ status. If we use the cut-off values for adults, almost one quarter of the infants were categorised as having low vitamin B_12_ (148–221 pmol/L) and 50% of the infants would have been classified as subclinical vitamin B_12_ deficient (<250 pmol/L).^(18)^ It is unclear whether the high prevalence of low vitamin B_12_ status in breastfed infants in our study is due to inappropriate cut-off values for this population or if breast milk is an insufficient source of vitamin B_12_. It has been suggested that the adequate range of serum cobalamin concentration in infants is lower than what is recommended for adults,^(1)^ while concerns have also been raised about vitamin B_12_ concentrations in breast milk and consequently low vitamin B_12_ status in breastfed infants.^(5,6)^

Plasma tHcy > 6.5 µmol/L, also denoted as the ‘vitamin B_12_ optimised’ tHcy cut-off, has been used in three studies among infants by the same authors.^(9,11,29)^ Two Norwegian RCTs reported improvements in motor function after intramuscular injection of 400 µg cobalamin for infants with elevated tHcy (> 6.5 µmol/L).^(11,29)^ However, the included infants had either low birth weight^(11)^ or feeding difficulties, minor neurologic symptoms, or developmental delay,^(29)^ limiting the generalisability of the findings. In addition, both studies had short follow-up periods, and the long-term effects could not be assessed. In a recently published study, 10% (*n* = 25) of 3–8-month-old infants were classified as suggestive vitamin B_12_ deficiency based on serum tHcy > 8 µmol/L, in combination with tremor or excessive sleep.^(37)^ In this prospective study, infants that scored below the 5th percentile on fine motor skills for the Age and Stages Questionaries (ASQ) had, on average, higher circulating tHcy concentrations compared to infants with normal scores. Another important finding of the latter study was that in 78% of the cases with tHcy concentration > 8 µmol/L, the infants’ parents did not report any vitamin B_12_ deficiency-related symptoms. Accordingly, the ‘vitamin B_12_-optimised’ tHcy cut-off value may appear to have a high sensitivity yet low specificity for symptomatic vitamin B_12_ deficiency. The results from the abovementioned studies build a strong case underscoring the need for RCTs in healthy infants investigating the short- and long-term effects of vitamin B_12_ supplementation.

Strengths of the present study included the measurement of both direct and functional biomarkers of vitamin B_12_ status in a population-based study of healthy infants. It is also a strength that we included participants at different ages of infancy, allowing breastfeeding status and biomarker concentrations to be evaluated independently of age. However, residual confounding factors may limit the interpretation of our findings. Other weaknesses of this study are the age difference across the breastfeeding groups, the relatively small sample size, and we did not have information about breastfeeding duration and when the feeding regime changed for each infant. Thus, it is possible we may not have had sufficient statistical power to measure the association between the biomarkers, breastfeeding, and age. In addition, the participants were not selected at random, which limits the generalisability. Furthermore, we did not analyse total serum holo-transcobalamin, the bioactive form of vitamin B_12_ which is often utilised in combination with other biomarkers to evaluate vitamin B_12_ status.^(18)^ Lastly, we did not measure maternal or breast milk vitamin status, nor did we assess infant dietary folate or vitamin B_12_ intake, which could have impacted the interpretation of the results.

### Conclusion

We found that none of the infants were folate deficient; however, low vitamin B_12_ status was prevalent and appeared to be more common in the younger breastfed infants compared to older weaned infants. The implications of low vitamin B_12_ status in infancy are unknown and require further investigation.
